# Risk of Accidental Falls Among Informal Caregivers

**DOI:** 10.1002/hsr2.71819

**Published:** 2026-03-22

**Authors:** Ayako Hiyoshi, Katja Fall, Scott Montgomery, Mikael Rostila, Alessandra Grotta

**Affiliations:** ^1^ Clinical Epidemiology and Biostatistics, School of Medical Sciences, Faculty of Medicine and Health Örebro University Örebro Sweden; ^2^ Department of Public Health Sciences Stockholm University Stockholm Sweden; ^3^ Integrative Epidemiology, Institute of Environmental Medicine Karolinska Institutet Stockholm Sweden; ^4^ Clinical Epidemiology Division, Department of Medicine Solna, Karolinska Institutet Stockholm Sweden; ^5^ Department of Epidemiology and Public Health University College London London UK; ^6^ Centre for Health Equity Studies Stockholm University/Karolinska Institutet Stockholm Sweden

**Keywords:** caregiving, falls, longitudinal study, socioeconomic position

## Abstract

**Background and Aims:**

Informal caregiving has increased over recent decades. Caregivers may face an increased risk of accidental falls because of care tasks or their consequences, such as fatigue. However, this association has not been investigated. Therefore, we aimed to examine whether giving personal care to someone at home increases fall risk.

**Methods:**

Using longitudinal repeated measures for adults aged over 50 years in 17 European countries, with biennial data collection in 2004–2017 (*N* = 51,132), we compared periods of caregiving to non‐caregiving for falls (outcome) using fixed‐effects logistic models, estimating odds ratios (OR) with 95% confidence intervals (95% CI) while controlling for measured time‐varying variables as well as unmeasured time‐invariant confounders. To shed light on mechanisms, we tested effect modification by sociodemographic characteristics, and examined whether fatigue, sleep problems, lower concentration, and changes in behaviour mediate the association.

**Results:**

Compared with the period of not giving care, the period of providing care was associated with higher fall risk (OR 1.19 [95% CI 1.05–1.35]). This association differed by baseline household income (below or above median). In higher‐income households, there was no statistically significant difference in fall risk between the period giving and not giving care (OR 1.07 [95% CI 0.90, 1.26]). In contrast, in lower‐income households, the caregiving period was associated with higher fall risk (OR 1.36 [95% CI 1.14–1.63]), which was equivalent to 29% (95% CI 12–46) increased probability of falls in caregiving periods. Fatigue, sleep problems, lower concentration, and behavioural changes jointly mediated 12% of the effect; thus, most of the effect of caregiving on falls is a direct effect.

**Conclusion:**

There was an increased fall risk among caregivers who provide personal care at home in lower‐income households. Fatigue and other consequences of caregiving mediated only small effects. Other factors, such as lack of equipment and living space, may relate to mechanisms.

## Summary

- Falls are often overlooked but are a common cause of injury that incurs substantial economic costs.

- Informal caregivers may face an increased risk of falls due to caregiving activities and their consequences, such as fatigue. However, their risk of falling has not been examined.

- This study found that caregivers' fall risk increases when providing personal care at home, but the increased risk was limited to caregivers in lower‐income households.

## 1 Introduction

Falls are often overlooked but are a common cause of injury. Most falls are non‐fatal, but they are associated with injury, functional decline [1], increased probability of a nursing‐home placement [2], and high healthcare costs [3, 4]. Even without injury, falls may lead to fear of falling and subsequent reduced mobility, which may lead to further negative health consequences [5, 6].

Individuals who provide unpaid care for a family member or others, known as informal caregivers, may face an increased risk of falls. However, only one cross‐sectional study so far has explored this association and reported a higher fall risk in daily caregivers [7]. Falls may occur in the act of caregiving because of, for example, balance loss while supporting the care recipient [8]. Consequences of caregiving, such as fatigue [9], sleep problems [10], reduced concentration [11, 12], changes in behavior [13], and poorer health may also elevate fall risk.

We examined whether informal caregiving increases fall risk. We used within‐individual comparisons to account for confounding from within‐individual time‐invariant characteristics, even unmeasured ones, in addition to measured confounders. To shed light on potential mechanisms, we tested whether socioeconomic and demographic characteristics modify the association and examined whether psychological and behavioral changes induced by caregiving mediate the association between caregiving and falls.

## 2 Methods

The Survey of Health, Ageing and Retirement in Europe (SHARE) is a longitudinal data with approximately biennial data collections since 2004 from a cohort of individuals aged 50 years or older [14]. We used data from participants in 17 countries (Spain, Italy, Greece, Portugal, Poland, Estonia, Czech Republic, Slovenia, France, Germany, Switzerland, Belgium, Austria, Luxembourg, the Netherlands, Denmark, and Sweden) who were non‐institutionalized, had complete data for relevant variables, and participated in at least two data collection waves among waves one, two, four, five, six, and seven (2004–2017) (Supporting Information S1: eText 1 for the data sources). The Swedish Ethical Review Authority approved the study (2024‐00741‐01).

### 2.1 Caregiving

We created a binary variable for co‐resident personal caregiving (yes/no) depending on whether the participants answered “yes” to a question: “Is there someone living in this household whom you have helped regularly during the last 12 months with personal care, such as washing, getting out of bed, or dressing? By regularly, we meant daily or almost daily for at least 3 months”.

#### 2.1.1 Falls

We created a binary variable to indicate whether the participant reported having fallen at least once during the previous 6 months (yes/no).

#### 2.1.2 Potential Confounders

As factors associated with caregiving [15, 16] and falls [17], we adjusted for the following time‐varying variables: age (years), attained education (primary, secondary, or tertiary levels) [18], employment status (yes/no), marital status (married/partnership, never married, divorced, or widowed), wave‐ and country‐specific household income deciles (1–10), household size (one, two, or ≥ three persons), self‐rated health (excellent, very good, good, fair, or poor), and the number of self‐reported chronic diseases (none, one, two, three, or four to eleven) (Supporting Information S1: eText 2 for the list of chronic diseases).

#### 2.1.3 Potential Effect Modifiers

We assessed whether the participants' baseline age ( ≤ 65 vs. > 65 years), sex, household income (below or above median), household size, self‐rated health (poor and fair vs. good to excellent), and type of welfare state (four groups [13, 19]) modify the association (Supporting Information S1: eText 2 for country groups).

#### 2.1.4 Potential Mediators

We examined psychological, behavioral, and physical health variables that were previously suggested to be affected by caregiving and that may also relate to falls [13, 17, 20]. Psychological aspects included: fatigue (having too little energy during the previous month [yes/no]); sleep problems (having trouble sleeping recently [yes/no]); and difficulty in concentration (having difficulty concentrating on, for example, television programmes or reading [yes/no]). Behavioral aspects included: physical activity (engaging in a moderate level activity such as walking more than once a week [yes/no]); leisure pursuits (participation in sports, social, educational or other kinds of club activities [yes/no]); drinking (having alcoholic beverages [yes/no]); and, body mass index (calculated using self‐reported height and weight [kg/m²]). The participant's self‐rated health was examined as a mediator (instead of treating it as a confounder) in a sensitivity analysis because falls may have affected health in the same wave.

### 2.2 Analysis

For descriptive analyses, we used the first observed survey wave (baseline) for each participant. We classified participants who reported caregiving or falling at least once during the follow‐up as “ever” caregivers or fallen, respectively, otherwise “never”.

We calculated fall rate by assuming that caregiving and falls were measured over 6 months in each survey wave, and that a report of a fall refers to one fall.

To investigate the association between caregiving and falls, we implemented fixed‐effect (also known as conditional) logistic models, estimating odds ratios (OR) with 95% confidence intervals (95% CI). We fitted two models, without and with all measured time‐varying potential confounders (Model 1 and 2, respectively). Based on the likelihood ratio tests' results, we modeled income and age with a quadratic term and BMI with quadratic and cubic terms. ORs from fixed‐effect models are based on within‐individual comparisons, automatically adjusting for within‐individual time‐invariant characteristics, measured and unmeasured, in addition to variables included in the model. The average percentage change in the probability of fall associated with caregiving was computed using the average (semi‐) elasticities [21, 22].

We examined whether caregivers' baseline age, sex, health, household income, household size and welfare state types modify the association between caregiving and falls by including interaction terms and performing likelihood ratio tests.

**Figure 1:**
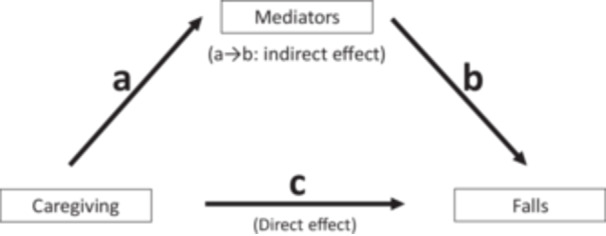
Schematic figure of hypothesized pathways linking caregiving to falls.

To quantify the extent to which psychological, behavioral, and health variables mediate the association between caregiving and falls, we decomposed the total effect of caregiving to fall into direct and indirect effects using the Karlson, Holm, and Breen (KHB) method with fixed‐effect logistic models [23]. The indirect effects are the effect of caregiving operated through the mediating factors in the model, that is, paths *a* to *b* in Figure 1 [23]. The remaining effect is the *direct effect* of caregiving to fall, the path *c*. Prior to fitting KHB models, the associations of the potential mediating factors with caregiving and falls were examined using the most‐adjusted fixed‐effect models.

In all the analyses, we estimated robust standard errors. Two sensitivity analyses were conducted. First, since the care recipient's health may also influence the caregiver's fall risk, we focused on caregiving for the partner and adjusted for their health (because the partner's health data were collected by SHARE). Second, we repeated KHB analyses by treating the caregiver's self‐rated health as a mediator.

We used Stata version 17 for analysis. *p* values < 0.05 were considered statistically significant.

## 3 Results

Out of 109,653 total participants, 70,960 responded to SHARE more than once (214,147 observations) (Supporting Information S1: eFigure 1). After excluding observations when individuals lived alone (46,191 observations) or with missing data in relevant variables (12,894 observations), 6469 participants had no longer multiple observations and were excluded. The remaining 148,593 observations from 51,132 participants were analyzed.

During the follow‐up from 2004 to 2017 (median number of available waves was three per participant [inter‐quartile range 2–3]), 8666 (17%) individuals provided care (Table 1). Among those who ever provided care, approximately 18% fell at least once, compared with 10% among those who never provided care. At baseline, caregivers were more likely to be female and older, have poorer health and disadvantaged socioeconomic characteristics, and live in Southern and Eastern Europe, compared to non‐caregivers.

**Table 1:** Distribution of participants according to whether they never or ever engaged in caregiving during the observation.

	Caregiving during the observation period	
	Never	Ever
Total (row %)	42,466 (83.1)	8666 (17.0)
	Freq.(col %)	Freq.(col %)
Falls during the observation period		
Never	38,080 (89.7)	7148 (82.5)
Ever	4386 (10.3)	1518 (17.5)
Characteristics at baseline survey (the earliest observed survey wave)		
Sex		
Men	21,204 (49.9)	3737 (43.1)
Women	21,262 (50.1)	4929 (56.9)
Household size		
2 persons	31,039 (73.1)	6092 (70.3)
3 or more persons	11,427 (26.9)	2574 (29.7)
Marital status		
Married/partnership	38,179 (89.9)	7615 (87.9)
Never married	930 (2.2)	285 (3.3)
Divorced	1640 (3.9)	298 (3.4)
Widowed	1717 (4.0)	468 (5.4)
Self‐rated health		
Excellent	3648 (8.6)	474 (5.5)
Very good	8389 (19.8)	1181 (13.6)
Good	16,501 (38.9)	2989 (34.5)
Fair	10,677 (25.1)	2823 (32.6)
Poor	3251 (7.7)	1199 (13.8)
Number of chronic diseases		
None	16,570 (39.0)	2792 (32.2)
One	13,369 (31.5)	2619 (30.2)
Two	7457 (17.6)	1748 (20.2)
Three	3333 (7.9)	922 (10.6)
4 to 11	1737 (4.1)	585 (6.8)
Education		
None/primary	9143 (21.5)	2537 (29.3)
Secondary	23,599 (55.6)	4656 (53.7)
Tertiary	9724 (22.9)	1473 (17.0)
Employment		
Employed	13,133 (30.9)	1683 (19.4)
Retired	22,864 (53.8)	5320 (61.4)
Sick/disabled	1253 (3.0)	338 (3.9)
Unemployed	1229 (2.9)	230 (2.7)
Homemaker	3987 (9.4)	1095 (12.6)
Country group		
Southern	9325 (22.0)	2299 (26.5)
Eastern	9212 (21.7)	2215 (25.6)
Bismarckian	18,064 (42.5)	3417 (39.4)
Scandinavian	5865 (13.8)	735 (8.5)
	Mean (SD)	Mean (SD)
Age (years)	64.7 (9.0)	67.1 (9.7)
Income (decile)	6.2 (2.7)	5.8 (2.6)

*Note:* In this table, participants who reported that they provided care or fell in at least one of survey waves during the follow‐up were defined as “ever” caregivers or fallen, respectively, otherwise “never”. Other variables were taken from the baseline (the earliest observed survey wave).

Abbreviations: Col%, column %; Freq, frequency; SD, standard deviation.

### 3.1 Caregiving and Falls

**Figure 2:**
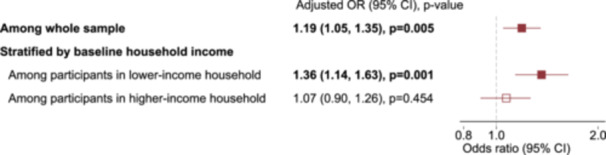
Fixed‐effects adjusted odds ratios for the associations between caregiving and falls for the whole sample and stratified by baseline household income. 95% CI: 95% confidence interval. Reference: non‐caregiving periods. The estimates are from Model 2, adjusting for within‐individual time‐invariant characteristics (such as sex, country of residence) as well as all available confounders as time‐varying variables: age, marital status, household size, self‐rated health, the number of chronic diseases, education, employment, and income decile as time‐varying variables. Age and income decile were used as continuous variables with quadratic terms. Other variables were used as categorical variables. Robust standard error was estimated.

Model 1, which adjusts for all within‐individual time‐invariant characteristics, showed that caregiving was associated with higher fall risk (OR 1.40 [95% CI 1.25, 1.57]) (Figure 2 and Supporting Information S1: eTable 1). Further adjustment for all measured time‐varying confounders attenuated the association, but a statistically significant higher risk remained (OR 1.19 [1.05, 1.35]).

The association between caregiving and fall risk differed by caregivers' baseline income decile (below or above median) (likelihood ratio test *p* value = 0.040) but not by their baseline age, sex, self‐rated health, or household size, or four country groups (Supporting Information S1: eTable 2). When data were stratified by household income at baseline, fall risk for caregiving in lower‐income households was OR 1.36 (1.14, 1.63) (Figure 2 and Supporting Information S1: eTable 1), whereas no association was observed in higher‐income households (OR 1.07 [0.90, 1.26]). In lower‐income households, the fall rate was approximately 5.6 per 100 individuals per 6 months in the period of non‐caregiving. The probability (semi‐elasticity) of fall increases by 29% (95% CI 12.4, 45.9) during caregiving periods, as estimated by using the most adjusted model.

### 3.2 Role of Potential Mediating Factors in Caregiving and Falls

Caregiving was associated with increased fatigue, trouble sleeping, and difficulty in concentration but reduced physical activity (Supporting Information S1: eTable 3), and these characteristics were in turn associated with higher fall risks (Supporting Information S1: eTable 4).

Based on the KHB method using the most adjusted fixed‐effect logistic models, the total effect of caregiving for falls is OR 1.41 [95% CI 1.17, 1.69] (*p* < 0.001) in lower‐income households. This effect was decomposed into direct (OR 1.35 [1.13, 1.62] *p* = 0.001) and indirect (OR 1.04 [1.03, 1.06] *p* < 0.001) effects. These estimates are interpreted as 12.2% of the total effect was an indirect effect operating through the mediating factors included in the model. Thus, the majority is a direct effect.

### 3.3 Sensitivity Analyses

First, elevated fall risk among caregivers in lower‐income households did not change after adjustment for the care recipient's (partner's) health (OR 1.38 [1.10–1.73]). Second, when the caregiver's self‐rated health was included as a mediator, the percentage of indirect effect increased to 28%, but the direct effect remained the largest (Supporting Information S1: eTable 5).

## 4 Discussion

In this study, providing personal care for someone at home increased fall risk, but only in lower‐income households. Psychological, behavioral, and health factors did not appear to play a major role as mediators in the association between caregiving and falls.

Our findings showing an increased fall risk associated with caregiving is consistent with results from a previous cross‐sectional study [7], but our study has several important methodological strengths. First, unlike the previous study, which compared groups of different individuals—caregivers with non‐caregivers, we used within‐individual comparisons. The estimates are thus the average change of fall risk when comparing caregiving periods to non‐caregiving periods while accounting for confounding rigorously [24, 25]. Second, unlike healthcare‐related data, in which only medically treated falls are identifiable, our data included any falls regardless of healthcare treatment. The accuracy of 12‐month recall of falls is relatively high [26]: the sensitivity and specificity are 80%–89% and 91%–95%, respectively [27]. Third, the frequencies of falls [26, 27] and caregiving [28, 29] may have been underestimated, and there may have been some residual confounding or other biases. However, if the mechanisms of biases and confounding structure were similar between higher‐ and lower‐income households, the fact that risk was specific only to lower‐income households implies that the finding was unlikely due to bias or confounding.

Considering the difference in caregiving‐related fall risk by household income and that the potential mediators we examined played a minor role, we can speculate that home environment may represent an important factor. Private homes may not be care‐friendly with limited space and aids, and inadequate lighting, furniture, and floor conditions [8]. Lower‐income households may have an even less suitable environment because of financial difficulties in adjusting the environment. Although our estimates are not confounded by pre‐existing disadvantages and health problems, the extra burden of caregiving added to the pre‐existing disadvantages may have resulted in increased falls in lower‐income households. Risks associated with factors intrinsic to caregivers or recipients, such as their age and health, may be difficult to modify, but risks related to the home environment can be prevented [30]. Further investigation into detailed mechanisms may provide valuable information.

Our study has some potential limitations. First, each survey wave is cross‐sectional, thus within‐wave temporal sequence of caregiving and falls is unclear. However, falling is unlikely to increase the likelihood of caregiving. Second, although the use of a large sample of several European countries is a strength of our study and implies that the findings may be generalizable to adults of similar ages in high‐income countries, the response rate of the SHARE survey was, on average, 51% across six survey waves used in the present study. If non‐participants had a higher likelihood of caregiving and falls than participants, we may have underestimated the association.

## 5 Conclusion

In this study of European adults aged 50 years and older, providing personal care for someone at home was associated with an increased risk of falls among caregivers in lower‐income households.

## Author Contributions

**Ayako Hiyoshi:** conceptualization, formal analysis, funding acquisition, investigation, methodology, project administration, resources, visualization, writing – original draft. **Katja Fall:** conceptualization, funding acquisition, methodology, writing – review and editing. **Scott Montgomery:** conceptualization, funding acquisition, methodology, writing – review and editing. **Mikael Rostila:** conceptualization, funding acquisition, methodology, writing – review and editing. **Alessandra Grotta:** conceptualization, data curation, funding acquisition, investigation, methodology, writing – review and editing.

## Funding

This study was supported by the Forskningsrådet om Hälsa, Arbetsliv och Välfärd (2019‐01236 and 2021‐00676) and Vetenskapsrådet (2022‐06397). The funders had no role in study design, data collection and analysis, decision to publish, or preparation of the manuscript.

## Conflicts of Interest

The authors declare no conflicts of interest.

## Impact Statement

We certify that this work is novel. Although family members often worry solely about the safety of those receiving care, and research has also focused on the risk to those receiving care, the concern about fall risk should extend to caregivers. This study found that caregivers' fall risk increases when informal caregivers provide personal care at home, but this risk was limited to caregivers in lower‐income households.

## Why does this paper matter?

Preventing falls among informal caregivers could be potentially achieved by raising awareness of this risk, as well as by modifying the home environment, and efforts could specifically target informal caregivers who provide care in lower‐income households.

## Transparency Statement

The corresponding author, Ayako Hiyoshi, affirms that this manuscript is an honest, accurate, and transparent account of the study being reported; that no important aspects of the study have been omitted; and that any discrepancies from the study as planned (and, if relevant, registered) have been explained.

## Supporting information


**eFigure 1:** Flowchart for analytical sample derivations. **eTable 1:** Fixed‐effects odds ratios for the associations between caregiving and falls. **eTable 2:** Likelihood ratio test *p* values for effect modification by sociodemographic characteristics. **eTable 3:** Odds ratios and coefficients for the likelihood of fatigue and other consequences when participants reported giving care. **eTable 4:** Odds ratios for the likelihood of falls when participants reported fatigue and other conditions. **eTable 5:** Fixed‐effects odds ratios for decomposing the OR for the risk of falls for caregiving in lower‐income households, using the Karlson, Holm, and Breen (KHB) method, additionally including the caregiver's self‐rated health as a mediator. **eText 1:** The list of sources of data. **eText 2:** Detailed information on the variables of (1) the number of chronic diseases, and (2) country groups.

## Data Availability

In this study, we used data from the Survey of Health, Ageing and Retirement in Europe (SHARE), which are publicly available to researchers upon registration. While the authors cannot publicly share the data used in this study, researchers interested in having access to the data can apply through the SHARE website (https://share‐eric.eu/data/data‐access).
