# Supplementary material for: Risk of Accidental Falls Among Informal Caregivers
Source: Health Sci Rep. 2026 Mar 22;9(3):e71819. doi: 10.1002/hsr2.71819 (PMC13098040; doi:10.1002/hsr2.71819)
Supplement: Supplementary file 1 — eFigure 1: Flowchart for analytical sample derivations. eTable 1: Fixed‐effects odds ratios for the associations between caregiving and falls. eTable 2: Likelihood ratio test p values for effect modification by sociodemographic characteristics. eTable 3: Odds ratios and coefficients for the likelihood of fatigue and other consequences when participants reported giving care. eTable 4: Odds ratios for the likelihood of falls when participants reported fatigue and other conditions. eTable 5: Fixed‐effects odds ratios for decomposing the OR for the risk of falls for caregiving in lower‐income households, using the Karlson, Holm, and Breen (KHB) method, additionally including the caregiver's self‐rated health as a mediator. eText 1: The list of sources of data. eText 2: Detailed information on the variables of (1) the number of chronic diseases, and (2) country groups. [file HSR2-9-e71819-s001.docx]

**ONLINE SUPPLEMENTARY MATERIAL**

**Contents**

[eText 2](#_Toc172737058)

[eText 1. The list of sources of data. 2](#_Toc172737059)

[eText 2. Detailed information on the variables of 1) the number of chronic diseases, and 2) country groups. 2](#_Toc172737060)

[eFigures 3](#_Toc172737061)

[eFigure 1. Flowchart for analytical sample derivations 3](#_Toc172737062)

[eTables 4](#_Toc172737063)

[eTable 1. Fixed-effects odds ratios for the associations between caregiving and falls 4](#_Toc172737064)

[eTable 2. Likelihood ratio test p-values for effect modification by sociodemographic characteristics 5](#_Toc172737065)

[eTable 3. Odds ratios and coefficients for the likelihood of fatigue and other consequences when participants reported giving care 6](#_Toc172737066)

[eTable 4. Odds ratios for the likelihood of falls when participants reported fatigue and other conditions 7](#_Toc172737067)

[eTable 5. Fixed-effects odds ratios for decomposing the OR for the risk of falls for caregiving in lower-income households, using the Karlson, Holm, and Breen (KHB) method, additionally including caregiver’s self-rated health as a mediator 8](#_Toc172737068)

Supplement to: Ayako Hiyoshi, Katja Fall, Scott Montgomery, Mikael Rostila, Alessandra Grotta. **Risk of accidental falls among informal caregivers.**

# eText

## eText 1. The list of sources of data.

- Börsch-Supan, A. (2022). Survey of Health, Ageing and Retirement in Europe (SHARE) Wave 1. Release version: 8.0.0. SHARE-ERIC. Data set. DOI: 10.6103/SHARE.w1.800
- Börsch-Supan, A. (2022). Survey of Health, Ageing and Retirement in Europe (SHARE) Wave 2. Release version: 8.0.0. SHARE-ERIC. Data set. DOI: 10.6103/SHARE.w2.800
- Börsch-Supan, A. (2022). Survey of Health, Ageing and Retirement in Europe (SHARE) Wave 4. Release version: 8.0.0. SHARE-ERIC. Data set. DOI: 10.6103/SHARE.w4.800
- Börsch-Supan, A. (2022). Survey of Health, Ageing and Retirement in Europe (SHARE) Wave 5. Release version: 8.0.0. SHARE-ERIC. Data set. DOI: 10.6103/SHARE.w5.800
- Börsch-Supan, A. (2022). Survey of Health, Ageing and Retirement in Europe (SHARE) Wave 6. Release version: 8.0.0. SHARE-ERIC. Data set. DOI: 10.6103/SHARE.w6.800
- Börsch-Supan, A. (2022). Survey of Health, Ageing and Retirement in Europe (SHARE) Wave 7. Release version: 8.0.0. SHARE-ERIC. Data set. DOI: 10.6103/SHARE.w7.800

## eText 2. Detailed information on the variables of 1) the number of chronic diseases, and 2) country groups.

1) The number of chronic diseases: The list of chronic diseases included in the counting was: heart attack, high blood pressure or hypertension, high blood cholesterol, stroke or cerebral vascular disease, diabetes or high blood sugar, chronic lung disease, cancer or malignant tumor, stomach or duodenal ulcer, peptic ulcer, Parkinson disease, cataracts, hip fracture and femoral fracture.

2) Country groups: Seventeen countries were classified into 4 groups: Southern (Spain, Italy, Greece, and Portugal), Eastern (Poland, Estonia, Czech Republic, and Slovenia), Bismarckian (France, Germany, Switzerland, Belgium, Austria, Luxembourg, and the Netherlands) and Scandinavian (Denmark and Sweden).

# eFigures

## eFigure 1. Flowchart for analytical sample derivations

**
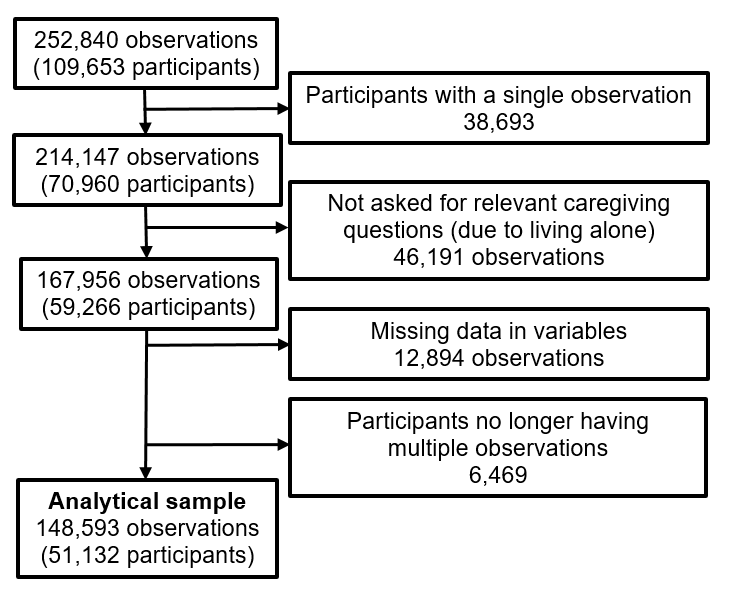
**

# eTables

## eTable 1. Fixed-effects odds ratios for the associations between caregiving and falls

|  | **Model 1** | | **Model 2** | |
| --- | --- | --- | --- | --- |
|  | **OR (95% CI)** | **P-value** | **OR (95% CI)** | **P-value** |
| **Whole sample** |  |  |  |  |
| Caregiving |  |  |  |  |
| No | Reference |  | Reference |  |
| Yes | 1.40 (1.25, 1.57) | <0.001 | 1.19 (1.05, 1.35) | 0.005 |
| **Stratified by baseline household income** |  |  |  |  |
| **Among individuals in lower-income households at baseline** | | | | |
| Caregiving |  |  |  |  |
| No | Reference |  | Reference |  |
| Yes | 1.63 (1.38, 1.93) | <0.001 | 1.36 (1.14, 1.63) | 0.001 |
| **Among individuals with higher-income households at baseline** | | | | |
| Caregiving |  |  |  |  |
| No | Reference |  | Reference |  |
| Yes | 1.22 (1.04, 1.44) | 0.014 | 1.07 (0.90, 1.26) | 0.454 |

OR: odds ratio.

95% CI: 95% confidence interval.

Reference: Non-caregiving periods.

Model 1: Adjusted for within-individual time-invariant characteristics through the model. Robust standard errors were estimated.

Model 2: Model 1+ all available confounders as time-varying variables: age, marital status, household size, self-rated health, the number of chronic diseases, education, employment and income decile as time-varying variables. Age and income decile were used as continuous variables with quadratic terms. Other variables were used as categorical variables.

Estimates are shown in Figure 2.

## eTable 2. Likelihood ratio test p-values for effect modification by sociodemographic characteristics

|  | **P-values** |
| --- | --- |
| Caregiving*Baseline household income decile (below or above median) | 0.040 |
| Caregiving*Baseline age (≤65 vs >65) | 0.129 |
| Caregiving*Sex | 0.205 |
| Caregiving*Baseline self-rated health (fair/poor vs good-excellent) | 0.509 |
| Caregiving*Baseline household size (2 vs 3 or more) | 0.847 |
| Caregiving*Country group (Southern, Eastern, Bismarckian or Scandinavian) | 0.602 |

Likelihood ratio test compared models with and without an interaction term between caregiving and household income, self-rated health, sex, age, household size and country groups. The models were adjusted for within-individual time-invariant characteristics as well as for time-varying confounders.

## eTable 3. Odds ratios and coefficients for the likelihood of fatigue and other consequences when participants reported giving care

|  | **OR (95% CI)** | **P-value** |
| --- | --- | --- |
| **Caregiving** |  |  |
| **Fatigue** | | |
| No | Reference |  |
| Yes (giving care) | 1.24 (1.16, 1.33) | <0.001 |
| **Trouble sleeping** | | |
| No | Reference |  |
| Yes (giving care) | 1.32 (1.23, 1.42) | <0.001 |
| **Difficulty in concentration** | | |
| No | Reference |  |
| Yes (giving care) | 1.28 (1.18, 1.39) | <0.001 |
| **Lack of physical activity** | | |
| No | Reference |  |
| Yes (giving care) | 0.88 (0.82, 0.95) | 0.001 |
| **Lack of leisure pursuits** | | |
| No | Reference |  |
| Yes (giving care) | 0.98 (0.90, 1.06) | 0.613 |
| **Drinking** | | |
| No | Reference |  |
| Yes (giving care) | 1.05 (0.97, 1.14) | 0.202 |
|  |  |  |
|  | **Coefficient (95% CI)** | **P-value** |
| **Body mass index** | | |
| No | Reference |  |
| Yes (giving care) | -0.05 (-0.10, 0.00) | 0.049 |

OR: odds ratio.

95% CI: 95% confidence interval.

ORs were obtained from fully adjusted fixed-effects (conditional) logistic models, and the coefficient was obtained from fixed-effects linear models.

Estimates are adjusted for all available confounders as time-varying variables: age, marital status, household size, self-rated health, the number of chronic diseases, education, employment and income decile using concurrent wave. Age and income decile were used as continuous variables with quadratic terms. Other variables were used as categorical variables. Robust standard errors were estimated.

## eTable 4. Odds ratios for the likelihood of falls when participants reported fatigue and other conditions

|  | **OR (95% CI)** | **P-value** |
| --- | --- | --- |
| **Fatigue** |  |  |
| No | Reference |  |
| Yes (fatigued) | 1.36 (1.26, 1.48) | <0.001 |
| **Trouble sleeping** |  |  |
| No | Reference |  |
| Yes (having trouble) | 1.35 (1.24, 1.47) | <0.001 |
| **Difficulty in concentration** |  |  |
| No | Reference |  |
| Yes (having difficulty) | 1.16 (1.06, 1.28) | 0.002 |
| **Lack of physical activity** |  |  |
| > once/week | Reference |  |
| ≤ once/week | 1.15 (1.06, 1.25) | 0.001 |
| **Lack of leisure pursuits** |  |  |
| No | Reference |  |
| Yes (lacked pursuits) | 0.95 (0.85, 1.05) | 0.301 |
| **Drinking** |  |  |
| No | Reference |  |
| Yes | 1.05 (0.95, 1.16) | 0.375 |
|  |  |  |
| **Body Mass Index (continuous)** | 1.01 (0.99, 1.03) | 0.179 |

OR: odds ratio.

95% CI: 95% confidence interval.

ORs were obtained from fully-adjusted fixed-effects (conditional) logistic models.

Estimates are adjusted for all available confounders as time-varying variables: age, marital status, household size, self-rated health, the number of chronic diseases, education, employment and income decile using concurrent wave. Age and income decile were used as continuous variables with quadratic terms. Other variables were used as categorical variables. Robust standard error was estimated.

## eTable 5. Fixed-effects odds ratios for decomposing the OR for the risk of falls for caregiving in lower-income households, using the Karlson, Holm, and Breen (KHB) method, additionally including caregiver’s self-rated health as a mediator

|  | **OR (95% CI)** | **P-value** |
| --- | --- | --- |
| Total effect | 1.51 (1.26, 1.81) | <0.001 |
| Direct effect | 1.35 (1.13, 1.62) | 0.001 |
| Indirect effect | 1.12 (1.09, 1.15) | <0.001 |
| Mediated percentage | 27.6% | |

95% CI: 95% confidence interval.

Estimates are adjusted for within-individual time-invariant and time varying characteristics (except for self-rated health). Robust standard error were estimated, as in Model 2 in Figure 2.

Total effect is the total effect of caregiving on fall risk.

Direct effect is the effect of caregiving on fall risk not through the mediators included in the model.

Indirect effect is the effect through the mediators included in the model: fatigue, trouble sleeping, difficulty in concentration, physical activity, lack of leisure pursuits, BMI, drinking and self-rated health. BMI was used as continuous variables with cubic terms.

Mediated percentage is calculated by KHB model, using scale-adjusted log ORs.
